# Sequestration and Distribution Characteristics of Cd(II) by* Microcystis aeruginosa* and Its Role in Colony Formation

**DOI:** 10.1155/2016/9837598

**Published:** 2016-09-29

**Authors:** Xiangdong Bi, Ran Yan, Fenxiang Li, Wei Dai, Kewei Jiao, Qixing Zhou, Qi Liu

**Affiliations:** ^1^Key Laboratory of Pollution Processes and Environmental Criteria, College of Environmental Science and Engineering, Nankai University, Ministry of Education, Tianjin 300071, China; ^2^Key Laboratory of Aquatic-Ecology and Aquaculture of Tianjin, Department of Fisheries Sciences, Tianjin Agricultural University, Tianjin 300384, China

## Abstract

To investigate the sequestration and distribution characteristics of Cd(II) by* Microcystis aeruginosa* and its role in* Microcystis* colony formation,* M. aeruginosa* was exposed to six different Cd(II) concentrations for 10 days. Cd(II) exposure caused hormesis in the growth of* M. aeruginosa*. Low concentrations of Cd(II) significantly induced formation of small* Microcystis* colonies (*P* < 0.05) and increased the intracellular polysaccharide (IPS) and bound extracellular polysaccharide (bEPS) contents of* M. aeruginosa* significantly (*P* < 0.05). There was a linear relationship between the amount of Cd(II) sequestrated by algal cells and the amount added to cultures in the rapid adsorption process that occurred during the first 5 min of exposure. After 10 d,* M. aeruginosa* sequestrated nearly 80% of 0.2 mg L^−1^ added Cd(II), while >93% of Cd(II) was sequestrated in the groups with lower added concentrations of Cd(II). More than 80% of the sequestrated Cd(II) was bioadsorbed by bEPS. The Pearson correlation coefficients of exterior and interior factors related to colony formation of* M. aeruginosa* revealed that Cd(II) could stimulate the production of IPS and bEPS via increasing Cd(II) bioaccumulation and bioadsorption. Increased levels of cross-linking between Cd(II) and bEPS stimulated algal cell aggregation, which eventually promoted the formation of* Microcystis* colonies.

## 1. Introduction

*Microcystis* blooms frequently occur in eutrophic freshwaters and their outbreaks always lead to water deoxygenation, microcystin pollution, and fish kill. During* Microcystis* blooms, algal cells aggregate largely on the surface of water and can be visible. Colony formation plays a vital role in the occurrence of* Microcystis* blooms [1]. It protects* Microcystis* cells from zooplankton grazing [2], viral or bacterial attack, and other potential negative environmental factors [3] and also provides a competitive advantage over other phytoplankton species [4]. However, how unicellular* Microcystis* cells aggregate into colonies exactly is not yet known. Previous studies have found that both biotic and abiotic factors, such as zooplankton grazing, nutritive salt, and microcystins, could stimulate* Microcystis* colony formation [2, 4]. Both zooplankton grazing and microcystins were reported to increase the amount of extracellular polysaccharides (EPS) and eventually stimulate aggregation of* Microcystis* cells [2].

Cyanobacterial blooms frequently occur in eutrophic waters with serious metal pollution [5, 6]. Cadmium (Cd), as a nonessential metal for biology, has become an important pollutant in natural freshwaters due to increasing use in industry, agriculture, and anthropogenic activities [7]. The negative effects of Cd on the environment have been recognized and it readily accumulates in living organisms. It was documented that cyanobacteria (*Synechocystis* sp. BASO670 and* Synechocystis* sp. BASO672) had high tolerance to Cd(II)-induced toxic effects, and Cd(II) could significantly stimulate EPS production in these two* Synechocystis* isolates [8]. In our previous study, we found there were significant differences in cadmium accumulation in four sizes of* Microcystis* colony [9] and cadmium accumulation decreased with increasing* Microcystis* colony sizes. We speculated that cadmium ions might play an important role in the early stage of* Microcystis* colony formation in natural waters [9].

To better predict, prevent, and control* Microcystis* blooms in natural waters, it is important to understand all the factors involved in triggering colony formation of* Microcystis* species. In this study, both sequestration and distribution characteristics of Cd(II) by toxic* M. aeruginosa* and physiological responses related to colony formation were investigated in laboratory conditions.

## 2. Materials and Methods

### 2.1. Cyanobacterial Culture

*M. aeruginosa* FACHB-905, a very common microcystin-producing* Microcystis* strain, which was provided by the Institute of Hydrobiology of China, can form large visible colonies with non-microcystin-producing* Microcystis* strains in natural waters.* M. aeruginosa* FACHB-905 were cultured in conical flasks containing sterilized BG11 medium (without EDTA) under a 12 light:12 dark cycle with a light density of 60 *μ*mol m^−2^ s^−1^ at 25°C. To reduce any effects related to minor differences in photon irradiance and to maintain homogeneity, the flasks were shaken slightly four times every day and rearranged randomly. Cultures were grown until the exponential growth phase.

### 2.2. Experimental Design

CdCl_2_ (Merck, Germany) was dissolved in distilled water to prepare a stock solution (20000 mg L^−1^). Based on the median effective concentration EC_50_-96 h = 0.383 mg L^−1^, determined by the method of Vanewijk and Hoekstra [10] of Cd(II) on* M. aeruginosa* obtained in preliminary experiments, Cd(II) was added to algal culture at an initial density of 5.473 × 10^6^ ind mL^−1^ to final concentrations of 0 (control), 0.0125, 0.025, 0.05, 0.1, 0.2, and 0.4 mg L^−1^, with three replicate flasks per concentration. Samples were removed from the cultures at 0, 5, 10, 20, 60, and 120 min and then daily until the 10th day of the experiment, to determine the algal cell density, colony numbers, bioadsorption and bioaccumulation characteristics of Cd(II), and bound extracellular polysaccharide (bEPS) and intracellular polysaccharide (IPS) contents of* M. aeruginosa*.

### 2.3. Effects of Cd(II) on Growth and Colony Formation of* M. aeruginosa*

Algal cell density and* M. aeruginosa* colonies were measured using a hemocytometer under a light microscope (×40). To reduce erroneous results, samples were taken 1 cm below the water surface with movement by a wide-mouth pipette, and the flasks were shaken up slightly 30 min before taking samples. The inhibition rate (IR), the decrease of intact algae in suspension, was calculated using the following formula: IR (%) = (*N*_0_ − *N*_*S*_)/*N*_0_ × 100, where *N*_0_ is algal cell density in the control (ind L^−1^) and* N*_*S*_ is algal cell density in the Cd(II)-added treatment (ind L^−1^) [11].* M. aeruginosa* were classified as unicellular, two-cell aggregation or colony (aggregation of ≥3 cells).

### 2.4. Determination of Bioadsorption and Bioaccumulation of Cd(II) by* M. aeruginosa*

Algal culture (500 mL) was centrifuged at 10,000 ×g for 10 min at 4°C, and the supernatant was dried to constant weight at 180°C to determine the content of Cd(II) in the medium (*μ*g L^−1^). The algal cell deposit was resuspended in 1.0 × 10^−3^ mol L^−1^ EDTA solution and then stirred with glass beads for 30 min to detach bEPS associated with the algal cells. After centrifugation at 10,000 ×g for 30 min at 4°C, the algal cell deposit was used to determine Cd(II) content accumulated inside algal cells (pg cell^−1^). The resulting supernatant was dried to constant weight at 180°C to determine Cd(II) content bioadsorbed on the algal cell walls. The resulting solid was precisely weighed (0.5 g) and transferred into a polytetrafluoroethylene, where 7 mL of HNO_3_ and 1 mL of H_2_O_2_ were added. The digester was sealed tightly and placed in a microwave digestion system (ETHOS One, Milestone, Italy). The procedure for microwave digestion is summarized as follows: the digester was heated from 25°C to 180°C over 10 min and then held for 25 min to digest the samples. After complete digestion, the digestion solution was evaporated to 2.5 mL, transferred to a 50 mL volumetric flask, and diluted with deionized water. A reagent blank was prepared using the same chemicals and digestion procedure as a comparison. Content of Cd(II) in collected samples was measured using Inductively Coupled Plasma-Atomic Emission Spectrometry (ICP-AES) using an ICP-9000 (N + M) (Thermo Jarrell Ash, USA), for which the detection limit of Cd is 0.01 mg kg^−1^.

### 2.5. Effects of Cd(II) on bEPS and IPS Production by* M. aeruginosa*

Algal culture (50 mL) was centrifuged at 5,000 ×g for 10 min at 4°C, and the cell pellet was washed with 1.0 × 10^−3^ mol L^−1^ EDTA solution using a glass rod. The resulting suspension was stirred with glass beads to detach bEPS associated with the algal cells. After centrifugation at 10,000 ×g for 30 min at 4°C, the algal cell pellet and supernatant were collected to determine IPS and bEPS contents (pg cell^−1^), respectively. To precipitate proteins from the supernatant, trichloroacetic acid (TCA) was added to a final concentration of 10% and the precipitated proteins were removed by centrifugation at 10,000 ×g for 20 min at 4°C. The clear supernatant was precipitated overnight at 4°C with six volumes of 95% ethanol. TCA precipitation and ethanol precipitation were repeated once. After centrifugation (12,000 ×g for 30 min at 4°C), the deposit obtained was dissolved in distilled water, dialyzed (3,600 mol wt cutoff tubing) against distilled water at 4°C for 24 h with 2 times, and then concentrated to 2 mL by rotary evaporation. The content of bEPS (pg cell^−1^) was estimated using the phenol-sulfuric acid method [12]. Algal cells were disrupted by freezing and thawing repeatedly to obtain IPS and then treated as for bEPS measurement.

### 2.6. Statistical Analysis

Results are expressed as means ± SD and were subjected to Fisher's least significant difference test (SPSS version 17.0) to determine significant differences (*P* < 0.05) among groups [13]. The Pearson correlation coefficient was examined by paired* t*-test (SPSS version 17.0) [14].

## 3. Results and Discussion

### 3.1. Effects of Cd(II) on Growth of* M. aeruginosa*

**Figure 1:**
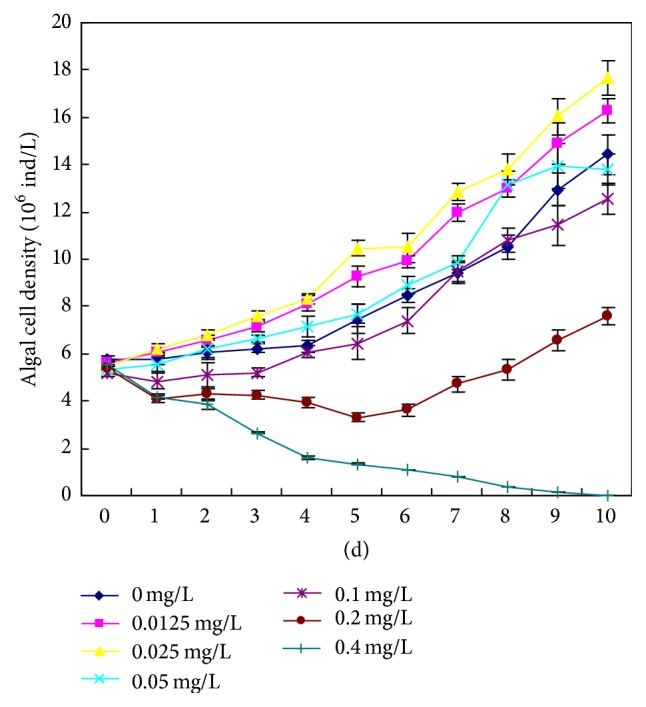
Effects of various concentrations of Cd(II) on algal cell density of* M. aeruginosa*.

**Figure 2:**
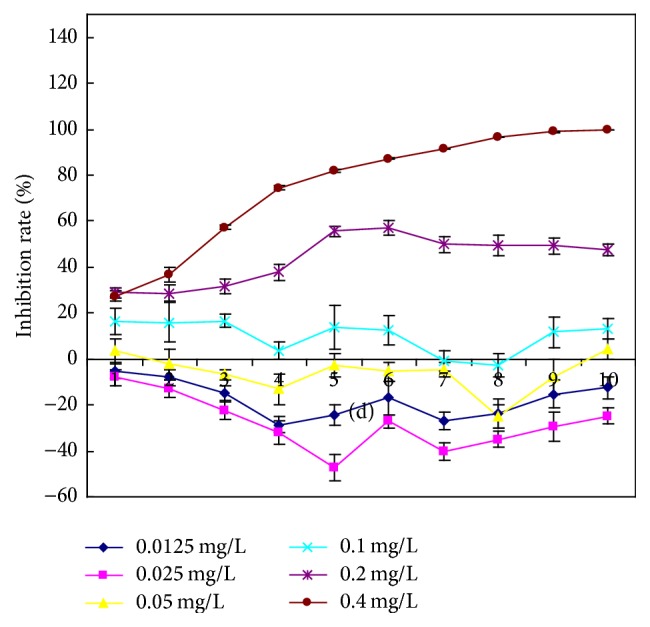
Effects of various concentrations of Cd(II) on the inhibition rate of* M. aeruginosa*.

The growth of* M. aeruginosa* FACHB-905 was affected in a Cd(II) concentration-dependent manner. With increasing exposure time,* M. aeruginosa* cell densities increased in low concentration Cd(II)-added groups (0.0125 and 0.025 mg L^−1^) and decreased in high-concentration Cd(II)-added groups (0.05, 0.1, 0.2, and 0.4 mg L^−1^) as compared with the control groups (Figure 1). These results indicated that Cd(II) had a dual effect (promotion and inhibition) on the growth of* M. aeruginosa*. Such an effect is described as hormesis, a dose-response relationship characterized by low-dose stimulation and high-dose inhibition [15]. Gong et al. found that inorganic arsenic could also cause hormesis in* M. aeruginosa* FACHB 905 [16]. In our experiment, high Cd(II) of concentrations inhibited the growth, possibly because the Cd(II) concentration exceeded the tolerance limit of* M. aeruginosa* cells, causing cell structure damage and disintegration. Among the tested concentrations, 0.4 mg L^−1^ Cd(II) exhibited the strongest inhibitory effects, with a 4-day IR of 74.752% and a 10-day IR of 99.992% (Figure 2). It is documented that* M. aeruginosa* PCC 7806 was susceptible to trace metal toxicity [17], while a* M. aeruginosa* strain isolated in the Czech Republic had high tolerance to 5.0–10.0 mg L^−1^ Cd(II) [18]. Therefore, it is possible that different strains of* M. aeruginosa* have different sensitivities to Cd(II)-induced toxicity.

### 3.2. Effects of Cd(II) on Colony Formation by* M. aeruginosa*

**Figure 3:**
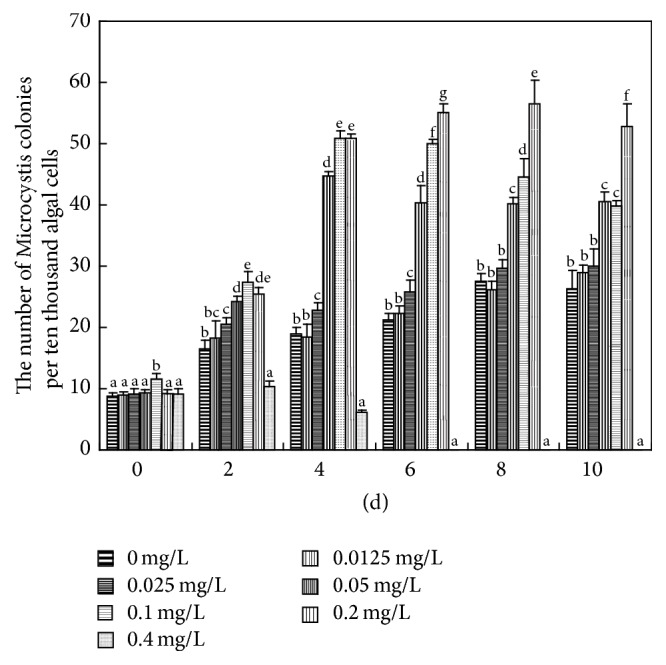
Effects of Cd(II) on colony formation by* M. aeruginosa*. Values sharing the same letters are not significantly different, whereas those marked with different letters are significantly different (*P* < 0.05).

Compared with colonial forms that predominate in natural conditions,* Microcystis* exists mainly as noncolonial (single and a few paired) cells in culture [19]. Colony formation of* M. aeruginosa* is a phenotypic response of single cells to environmental stress [3]. Colonial* Microcystis* had higher endurance to heavy metal-induced stress than noncolonial* Microcystis* [20]. Our results show that three concentrations of Cd(II) (0.05, 0.1, and 0.2 mg L^−1^) significantly induced* Microcystis* colony formation (*P* < 0.05), and 0.2 mg L^−1^ Cd(II) had the highest inductive effect (Figure 3). The proportion of* Microcystis* colonies was significantly decreased in the 0.4 mg L^−1^ Cd(II)-added group after 4 d, and no* Microcystis* colonies could be observed under the microscope after 6 d, which might result from the strong inhibitory effects of 0.4 mg L^−1^ Cd(II) on the growth of* M. aeruginosa* (Figures 1, 2, and 3). No colonies with five or more cells were observed during the whole experimental period. Unlike Fe, Al, Mn, Pb, and Cr, Cd was the only heavy metal element accumulated much higher in small* Microcystis* colony than in middle, large, and super-large colonies in natural waters [9]. These observations indicate that Cd(II) may play an important role in the early stage of* Microcystis* colony formation in natural waters [9].

### 3.3. Sequestration and Distribution Characteristics of Cd(II) by* Microcystis* Cells

When* Microcystis* cells were exposed to Cd(II), a rapid adsorption process occurred in the first 5 min. There was a linear relationship between Cd(II) sequestrated by algal cells (including Cd(II) bioadsorbed on bEPS and bioaccumulated inside cells) and the added Cd(II) concentrations during this time period (*y* = 8.5326*x* − 8.664; *R* ^2^ = 0.9864). More chances for metal ions to combine with cation-chelating binding sites in bEPS were provided with the increasing Cd(II) concentrations [21, 22]. Once Cd(II) was bioadsorbed onto the external cell wall, an internal bioaccumulation process would begin immediately [23]. Use of different strains of* M. aeruginosa* might lead to a longer adsorption process (10 min) [24]. In our experiment, there was also a slow adsorption process following the initial 5 min rapid adsorption, suggesting that Cd(II) was removed via interactions with functional groups on the cell surface [8, 17].

**Figure 4:**
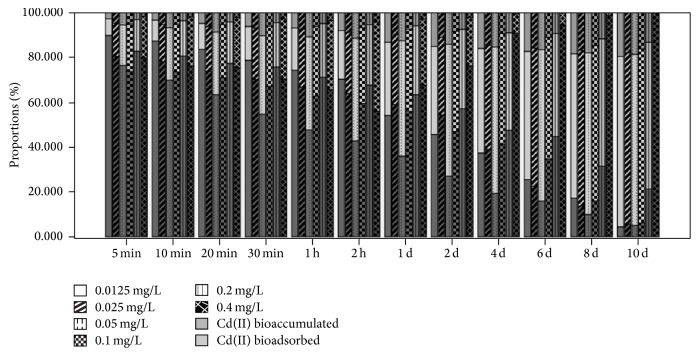
Changes in the proportions of Cd(II) bioadsorbed by bEPS and bioaccumulated inside algal cells and of Cd(II) in the culture medium for* M. aeruginosa* exposed to various concentrations of Cd(II).

**Figure 5:**
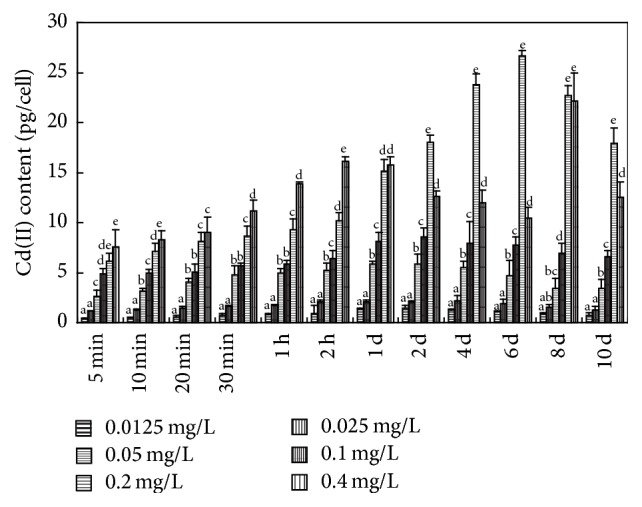
Cd(II) content bioaccumulated inside algal cells. Values sharing the same letters are not significantly different, whereas those marked with different letters are significantly different (*P* < 0.05).

**Figure 6:**
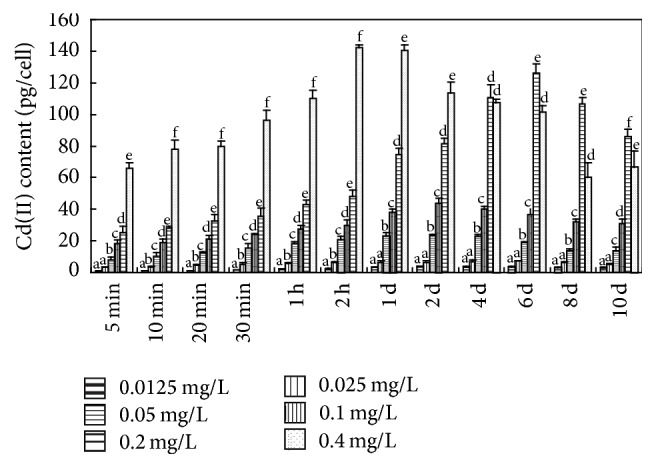
Cd(II) bioadsorbed by bEPS. Values sharing the same letters are not significantly different, whereas those marked with different letters are significantly different (*P* < 0.05).

On longer Cd(II) exposure the proportion of Cd(II) sequestrated in* Microcystis* cells increased, reaching >93% at the end of the 10th day in the groups exposed to ≤0.1 mg L^−1^ Cd(II) (Figure 4).* M. aeruginosa* sequestrated >80% of the 0.2 mg L^−1^ added Cd(II). We suggest that when 0.2 mg L^−1^ Cd(II) was added, Cd(II) sequestration by* M. aeruginosa* cells reached its saturation level. Considering the sequestration Cd(II) in* M. aeruginosa*, >80% was bioadsorbed by bEPS and <20% was bioaccumulated inside algal cell (Figures 5 and 6), which is consistent with the results of Parker et al. [25] and Ozturk et al. [8].

### 3.4. Effects of Cd(II) on bEPS and IPS Production by* M. aeruginosa*

**Figure 7:**
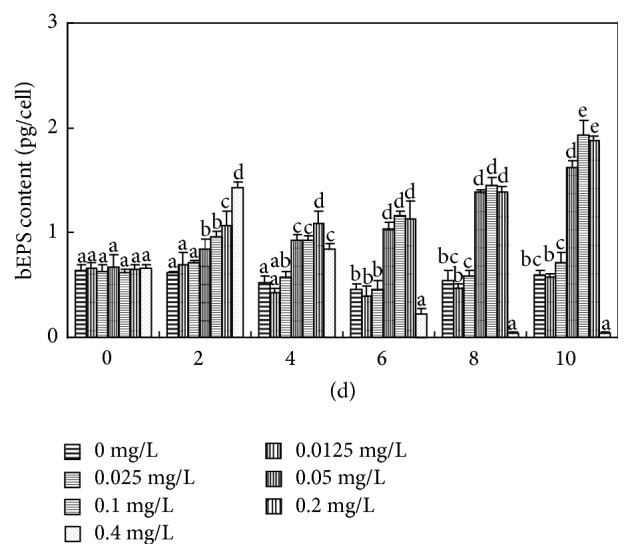
Effects of various concentrations of Cd(II) on the bEPS content of* M. aeruginosa* (pg/cell). Values sharing the same letters are not significantly different, whereas those marked with different letters are significantly different (*P* < 0.05).

**Figure 8:**
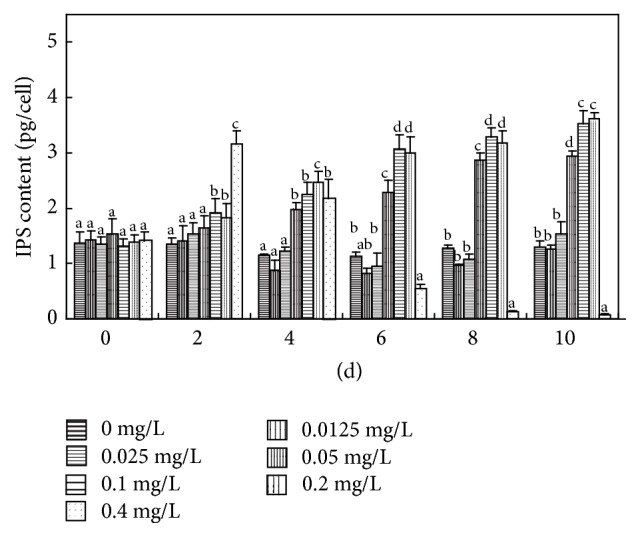
Effects of various concentrations of Cd(II) on the IPS content of* M. aeruginosa *(pg/cell). Values sharing the same letters are not significantly different, whereas those marked with different letters are significantly different (*P* < 0.05).

Under normal physiological conditions, polysaccharide produced in cells can be partially secreted to form EPS. The cyanobacterial EPS can be divided into two main groups: polysaccharides bound to the cell surface (bEPS) and soluble polysaccharides released into the surrounding environment (soluble extracellular polysaccharide, sEPS) [26]. Bound extracellular polysaccharide, referred to as sheath, capsule, or slime, plays an important role in the attachment of* M. aeruginosa* cells into colonies and in protecting cells from unfavorable environmental conditions [26]. Compared to the control, no significant changes in IPS content were observed in* M. aeruginosa* exposed to 0.0125 and 0.025 mg L^−1^ Cd(II) throughout the experiment, and a similar result was obtained for bEPS for the first 6 days (Figures 7 and 8), because* M. aeruginosa* in long-term laboratory culture responded only slowly to slight environmental disturbance [27]. Higher concentrations of Cd(II) (0.05, 0.1, and 0.2 mg L^−1^) significantly and simultaneously increased IPS and bEPS levels throughout the experimental period (*P* < 0.05) (Figures 7 and 8). IPS and bEPS contents changed in the same way as each other after Cd(II) exposure and had a high Pearson correlation coefficient throughout the experiment (Table 1), suggesting* Microcystis* cells could respond positively to Cd(II) stress by secreting organic substances (mainly polysaccharides) via active transport [28] (Figures 7 and 8). It is also worth noting that Cd(II) could significantly stimulate bEPS production of* M. aeruginosa* at relatively low concentrations, while the stimulatory activities of Pb(II) were observed at much higher concentrations [29]. Therefore, we speculate that Cd(II) at low concentration could promote the early formation of* Microcystis* colonies by stimulating bEPS production in natural waters.

### 3.5. Role of Cd(II) in Colony Formation of* M. aeruginosa*

The Pearson correlation coefficients of exterior and interior factors related to colony formation by* M. aeruginosa* are shown in Table 1. With increasing concentrations of Cd(II) added to the culture medium, Cd(II) bioadsorption onto bEPS and Cd(II) bioaccumulation inside algal cells significantly increased. Increased Cd(II) bioaccumulation inside algal cells stimulated more IPS production. We suggest that, through active transport [28], more IPS was secreted outside the cells, leading to an increased chance of cross-linking between Cd(II) and bEPS [30]. Algal cell aggregation was stimulated by this increased cross-linking, which eventually promoted the formation of* Microcystis* colonies.

## 4. Conclusions

*M. aeruginosa* was very sensitive to Cd(II) compared to other heavy metal ions and adsorbed >93% of the Cd(II) in the culture medium when exposed to ≤0.1 mg L^−1^ Cd(II). Among the sequestrated Cd(II) in* M. aeruginosa*, >80% was bioadsorbed by bEPS, and <20% was bioaccumulated inside algal cells. Under the stress of Cd(II),* M. aeruginosa* increased the production of IPS and bEPS, which could significantly promote the formation of small colonies. Notably, this phenomenon occurred at low Cd(II) concentrations, close to those found in natural waters. Moreover, we have found that Pb(II) in the tolerance range of* M. aeruginosa* could stimulate bEPS production, which promoted colony formation [29]. Therefore, heavy metals with different stimulatory effects on different stages of the formation of* Microcystis* colonies might be one factor that contributes to the occurrence of* M. aeruginosa* blooms in natural conditions.

## Figures and Tables

**Table 1:** Pearson correlation coefficients of exterior and interior factors related to the colony formation of *M. aeruginosa*.

Time	Relationship of initial Cd(II) concentrations with amounts of Cd(II) bioabsorbed on bEPS per unit algal cell	Relationship of initial Cd(II) concentrations with amounts of Cd(II) bioaccumulated inside per unit algal cell	Relationship of amounts of Cd(II) bioabsorbed per unit algal cell with amounts of bEPS per unit algal cell	Relationship of amounts of Cd(II) bioaccumulated inside per unit algal cell with amounts of IPS per unit algal cell	Relationship of bEPS per unit algal cell with IPS per unit algal cell	Relationship of initial Cd(II) concentrations with *Microcystis* colony formation
2 d	0.994^*∗∗*^	0.977^*∗∗*^	0.897^*∗∗*^	0.513^*∗*^	0.891^*∗∗*^	0.724^*∗∗*^
4 d	0.988^*∗∗*^	0.980^*∗∗*^	0.829^*∗∗*^	0.821^*∗∗*^	0.985^*∗∗*^	0.823^*∗∗*^
6 d	0.976^*∗∗*^	0.975^*∗∗*^	0.667^*∗∗*^	0.732^*∗∗*^	0.981^*∗∗*^	0.914^*∗∗*^
8 d	0.978^*∗∗*^	0.978^*∗∗*^	0.628^*∗∗*^	0.682^*∗∗*^	0.990^*∗∗*^	0.955^*∗∗*^
10 d	0.987^*∗∗*^	0.986^*∗∗*^	0.737^*∗∗*^	0.795^*∗∗*^	0.997^*∗∗*^	0.933^*∗∗*^

^*∗∗*^Correlation is significant at the 0.01 level (two-tailed). ^*∗*^Correlation is significant at the 0.05 level (two-tailed).

## Abbreviations

IPS: Intracellular polysaccharide
bEPS: Bound extracellular polysaccharide
sEPS: Soluble extracellular polysaccharide
EPS: Extracellular polysaccharide
IR: Inhibition rate
ICP-AES: Inductively Coupled Plasma-Atomic Emission Spectrometry.
